# Genome-wide identification of the ABCB gene family in melon (*Cucumis melo* L.) and their expression during axillary bud development

**DOI:** 10.3389/fpls.2026.1770152

**Published:** 2026-04-22

**Authors:** Jun Lai, Lin Zhang, Yu Sun, Jiayu Wang, Kexin Kong, Yuanzuo Lv

**Affiliations:** Hebei Key Laboratory of Horticultural Germplasm Excavation and Innovative Utilization, College of Horticulture Science & Technology, Hebei Normal University of Science & Technology, Qinhuangdao, Hebei, China

**Keywords:** ABCB, axillary bud development, *Cucumis melo*, gene expression, IAA

## Abstract

**Introduction:**

Melon (*Cucumis melo* L.) is an important economic crop, yet its cultivation relies heavily on manual pruning to regulate branching, which limits melon industrialization. Branching originates from axillary bud development, a process regulated by polar auxin transport, in which ABCB proteins serve as key auxin transporters. However, systematic identification of the ABCB gene family in melon and the functional roles of its members in axillary bud development have not been systematically elucidated.

**Methods:**

We performed a genome-wide identification of the ABCB gene family from the latest melon genome using bioinformatics approaches. We systematically analyzed the physicochemical properties and other characteristics of the identified *CmABCB* genes. Additionally, RNA sequencing (RNA-seq) and qRT-PCR were employed to investigate the expression patterns of these genes in axillary buds under IAA treatment.

**Results:**

Through this genome-wide identification, we identified a total of 39 *CmABCB* genes in the melon genome. Phylogenetic analysis classified them into five subgroups, with Group I being unique to melon. Collinearity analysis revealed a close evolutionary relationship between melon and cucumber. Expression profiling revealed that several genes responded specifically to IAA treatment. Notably, *CmABCB14*, *CmABCB20*, and *CmABCB21* exhibited a statistically significant positive correlation between their expression levels and IAA concentration.

**Discussion:**

Collectively, these findings suggest that *CmABCB14*, *CmABCB20*, and *CmABCB21* may function as auxin efflux carriers that suppress axillary bud growth. This study therefore provides a theoretical basis for understanding the molecular mechanisms underlying branching regulation in melon and offers a valuable framework for the precise improvement of plant architecture and the reduction of production costs through molecular breeding.

## 1 Introduction

Melon (*Cucumis melo* L.), a member of the *Cucurbitaceae* family, is widely cultivated worldwide for its unique flavor and aroma. With an annual production exceeding 27 million tons in 2019, it is a crop of significant economic value (Zhao et al., 2019; Wang et al., 2022). Originating from Africa, melon is rich in nutrients such as vitamins, minerals, and dietary fiber (Sebastian et al., 2010; Tian et al., 2024). However, melon cultivation demands considerable manual labor input for pruning, which is essential to regulate branching and balance vegetative and reproductive growth. This labor-intensive pruning process significantly elevates production costs and thereby constrains the scaling-up of melon industrial cultivation.

Transcription factors play a crucial role in shoot branching, and numerous branching-related genes have been functionally characterized in various plant species. For instance, in apple, *MdWUS2* promotes shoot branching by inhibiting the activity of *MdTCP12* (Li et al., 2021). Overexpression of *SlTCP26* enhances shoot branching in tomato, while exogenous application of IAA repress *SlTCP26* expression to inhibit tomato bud outgrowth (Wei et al., 2021). In cucumber, *CsTIE1* directly interacts with *CsAGL16* and positively regulates shoot branching via the CsAGL16–CsCYP707A4 pathway (Chen et al., 2025). *GhCUC2* interacts with *GhBRC1* to inhibit cotton branching by increasing local abscisic acid (ABA) accumulation (Zhan et al., 2021). Although diverse shoot branching regulatory mechanisms have evolved across plant species, the molecular basis underlying shoot branching regulation in melon, particularly the key genes and their regulatory networks, has not been systematically elucidated to date. This knowledge gap hinders the development of molecular breeding strategies to optimize plant architecture and reduce labor input. Therefore, systematic identification and functional characterization of branching-related genes in melon are urgently needed to address the labor-intensive pruning challenge and promote the sustainable development of the melon industry (Wang et al., 2024).

Shoot branching directly affects plant architecture and exerts significant impacts on crop yield and quality (Mathan et al., 2016). During its development, axillary meristems formed in leaf axils further differentiate into axillary buds composed of leaf primordia and apical meristems. At this stage, axillary buds either enter dormancy or break dormancy to develop into lateral branches, a process tightly regulated by endogenous and exogenous signals (Li et al., 2024). Plant hormones play crucial regulatory roles in axillary bud growth: cytokinins (CK) promote axillary bud growth, while auxin (IAA), abscisic acid (ABA), and strigolactone (SL) exert inhibitory effects. The mechanisms by which these hormones regulate axillary bud have been well characterized in model plants (Barbier et al., 2019). As one of the earliest discovered plant hormones, auxin exhibits polar transport characteristics from the apex to the base and plays a pivotal role in various aspects of plant growth and development. Its role in regulating axillary bud growth has been well documented (Yang et al., 2020; Bao et al., 2024). Endogenous IAA levels are co-regulated by its synthesis, transport, and signal transduction processes, which in turn modulate axillary bud dormancy and outgrowth (typically inhibiting bud break). Intercellular IAA transport is primarily mediated by three types of auxin transporters: PIN-formed efflux carriers, ATP-binding cassette subfamily B (ABCB) transporters, and AUX1/LAX influx proteins (Carraro et al., 2012; Hammes et al., 2022).

Based on domain composition, ABCB family members are classified into two types: half-size transporters, which harbor one transmembrane domain (TMD, for substrate recognition and transmembrane transport) and one nucleotide-binding domain (NBD, for ATP binding and hydrolysis), and full-size transporters, which contain two TMD-NBD units (Hollenstein et al., 2007; Verrier et al., 2008). In *Arabidopsis thaliana*, eight *ABCB* genes have been identified as key regulators of auxin transport. Among them, *AtABCB1* and *AtABCB19*, as the earliest identified members of this family, both participate in auxin efflux (Geisler et al., 2005). *AtABCB4* enhances auxin efflux activity to reduce intracellular auxin levels in root hair cells, thereby inhibiting root hair elongation, though its potential role in axillary bud regulation remains unreported (Cho et al., 2008). *AtABCB6* and *AtABCB20* exhibit strong functional redundancy in auxin transport (Zhang et al., 2018). *AtABCB21* bidirectionally mediates auxin transmembrane transport in response to cytoplasmic auxin concentrations (Kamimoto et al., 2012). The ABCB gene family also plays crucial roles in other plant species. *OsABCB4* is involved in auxin efflux and regulates grain length and weight in rice (Du et al., 2025), while *MdABCB1* and *MdABCB19* participate in auxin efflux and indirectly modulate tree height via shoot elongation regulation in apple (Ma and Han, 2016). *SlABCB4* promotes auxin distribution in tomato fruits (Ofori et al., 2018), though the roles of these ABCB homologs in shoot branching remain uncharacterized.

To date, the ABCB gene family in melon genome has not been systematic identified, and the regulatory roles of its members in axillary bud development remain unreported. Therefore, based on the latest *Cucumis melo* genome (Garcia-Mas et al., 2012), we performed genome-wide identification of ABCB family members in melon and systematically analyzed their conserved motifs, gene structures, promoter *cis*-acting elements, chromosomal localizations, and phylogenetic relationships using bioinformatics approaches. Additionally, we determined the transcriptional profiles of *CmABCB* genes in axillary bud dormancy break and outgrowth under exogenous IAA treatment. The results of this study preliminarily reveal the regulatory roles of *CmABCB* genes in melon axillary bud development, providing a theoretical basis for optimizing melon plant architecture via molecular breeding and thereby reducing input associated with manual pruning.

## 2 Materials and methods

### 2.1 Identification and basic characterization of the melon ABCB gene family

Genome data of melon and cucumber were downloaded from the CuGenDB database (http://cucurbitgenomics.org/), and Hidden Markov Models of the ABC transporter (PF00005) and ABC transmembrane (PF00664) domains were retrieved from the Pfam database (http://pfam.xfam.org/). Using TBtools (Chen et al., 2020), protein sequences were extracted from the melon genome, and based on HMMER 3.1 (Yue et al., 2015), melon protein sequences containing both ABC transporter (PF00005) and ABC transmembrane (PF00664) domains were preliminarily screened. These sequences were then subjected to homology alignment with 28 AtABCB proteins obtained from the *A. thaliana* genome database TAIR (https://www.arabidopsis.org/) using the “Blast Compare Two Seq [Sets]” tool in TBtools, with the maximum number of target sequences and E-value set to 2 and 1e-5, respectively. The resulting melon ABCB candidate sequences were aligned against the NCBI “UniProtKB/Swiss-Prot (swissport)” database (Yang et al., 2021b), the most similar sequences were selected based on the smallest Q-value, and sequences that were too short or incorrectly annotated were removed, ultimately yielding 39 CmABCB family genes. The amino acid composition, molecular weight, and theoretical isoelectric point (pI) of CmABCB proteins were analyzed using ExPASy (http://web.expasy.org/protparam/) (Artimo et al., 2012), Cell-PLoc 2.0 (http://www.csbio.sjtu.edu.cn/bioinf/Cell-PLoc-2/) was used to predict their subcellular localization (Chou and Shen, 2010), and TMHMM 2 (https://services.healthtech.dtu.dk/services/TMHMM-2.0/) was employed to predict their transmembrane domains. The three-dimensional structures of the proteins were predicted using SWISS-MODEL (https://swissmodel.expasy.org/), and SSRs in the genomic sequences of the *CmABCB* genes were identified using TBtools SSRminer. The workflow for identification and basic characterization of the CmABCB gene family is shown in **Supplementary Figure S1**.

### 2.2 Phylogenetic analysis

To investigate the phylogenetic relationships of ABCB proteins between melon and *A. thaliana*, ClustalW was used for multiple sequence alignment of the identified melon ABCB proteins. MEGA11 (Tamura et al., 2021) was employed to construct a phylogenetic tree using the neighbor-joining method with bootstrap values set to 1000 based on the ABCB protein sequences from both melon and *A. thaliana*. The generated phylogenetic tree was visualized and enhanced using the online tool iTOL (https://itol.embl.de/) (Shang et al., 2025).

### 2.3 Analysis of motifs, domains, and gene structures

Conserved motif prediction of melon ABCB protein sequences was performed using the online MEME tool (https://meme-suite.org/meme/) with the number of motifs set to 10 (Bailey et al., 2009). Batch conserved domain analysis was conducted via the NCBI CDD online tool (https://www.ncbi.nlm.nih.gov/cdd/) (Lu et al., 2020), and the positions and numbers of exons and introns in genes were extracted from the melon genome GFF file. The gene structures, conserved motifs, and protein conserved domains of melon ABCB genes were visualized using TBtools.

### 2.4 Chromosomal localization and collinearity analysis

Based on the melon genome annotation file, the chromosomal positions of the 39 identified *CmABCB* genes were extracted and filtered. The chromosomal locations of the *CmABCB* genes were visualized using TBtools, their distribution was analyzed, and the genes were renamed according to their order on the chromosomes (Yang et al., 2021a). Collinearity analysis was performed using TBtools.

### 2.5 GO and KEGG enrichment analysis

GO and KEGG enrichment analyses were performed using TBtools with the melon whole-genome protein set as background. A corrected p-value (FDR) < 0.05 was considered significant. GO and KEGG annotations were obtained via eggNOG-mapper. Visualization was carried out using TBtools built-in functions.

### 2.6 Cis-acting element analysis

The promoter sequences spanning 2,000 bp upstream of the ATG start codon of the 39 *CmABCB* genes were extracted using TBtools. Putative *cis*-acting elements were identified in these promoter sequences through the PlantCARE online tool (http://bioinformatics.psb.ugent.be/webtools/plantcare/html/) (Lescot, 2002). *Cis*-acting elements associated with phytohormone response, growth and development, as well as abiotic and biotic stresses were subsequently selected and analyzed. These elements were classified into functional categories according to relevant literature (Mengarelli and Zanor, 2021; Xue et al., 2023). TBtools was employed to quantify and visualize the results.

### 2.7 Expression profiling of *CmABCB* genes in axillary buds during bud induction and qRT-PCR validation

Melon cultivar X055 was used as the plant material. Germinated seeds were sown in 50-cell plug trays filled with a mixture of plant ash and vermiculite (volume ratio of 3:1). When seedlings reached the two true leaves and one heart leaf stage, they were transplanted to a greenhouse. For the axillary bud hormone assay, 27 uniform melon seedlings were randomly divided into three groups and sprayed with distilled water (control, CK), 20 mg/L IAA, or 50 mg/L IAA. These concentrations, establishing a gradient from moderate to strong regulation, were selected based on preliminary experiments and studies of shoot branching in tomato (Wei et al., 2021) to capture a broad range of *CmABCB* transcriptional responses. Solutions were uniformly applied to axillary buds until runoff, with treatments administered on days 0, 3, 7, and 14 (four applications in total). To link transcriptomes to phenotypes, we monitored the elongation of axillary buds at the 5th–11th nodes (all dormant at Day 0): exogenous IAA significantly promoted bud outgrowth, with their average lengths similar at Day 3 (CK: 0.32 cm; IAA-treated groups: 0.31 cm), diverging at Day 7 (0.81 vs. 1.49 cm), and showing more pronounced divergence by Day 14 (4.91 vs. 8.25 cm; **Supplementary Figure S2**), thus ensuring sampling captured a defined rapid-elongation stage under IAA promotion. At 24 hours after the final treatment (day 14), axillary buds of melon X055 were sampled, frozen in liquid nitrogen, and stored at -80 °C until analysis. Transcriptome sequencing was entrusted to Shanghai Bioprofile Biotechnology Co., Ltd. (Shanghai, China), including strand-specific library construction and Illumina platform sequencing. Subsequently, the obtained high-quality sequences were aligned to the melon reference genome, and the FPKM values of each gene were calculated as normalized expression levels based on the alignment results.

Based on the FPKM values from the transcriptome data, we selected nine candidate *CmABCB* genes that showed significant expression differences among different IAA treatments for qRT-PCR validation. Total RNA was extracted from melon axillary buds using an RNA extraction kit (Vazyme, Nanjing, China) following the manufacturer’s instructions. Reverse transcription PCR (RT-PCR) was performed using an all-in-one mix kit (Vazyme, Nanjing, China). *CmActin* was selected as a stably expressed internal control, and all primers (sequences provided in **Supplementary Table S1**) were synthesized by Sangon Biotech (Shanghai, China). qRT-PCR was carried out using SYBR Green PreMix Plus (TIANGEN, Beijing, China) to determine the transcript levels of *CmABCB*s. The reaction protocol consisted of an initial step at 95 °C for 15 min, followed by 40 cycles of 95 °C for 10 s and 60 °C for 32 s. The relative expression levels of the genes were calculated using the 2^−ΔΔCT^ method. All experiments were performed with three biological replicates and three technical replicates.

## 3 Results

### 3.1 Identification and basic characterization of the ABCB gene family in melon

**Figure 1:**
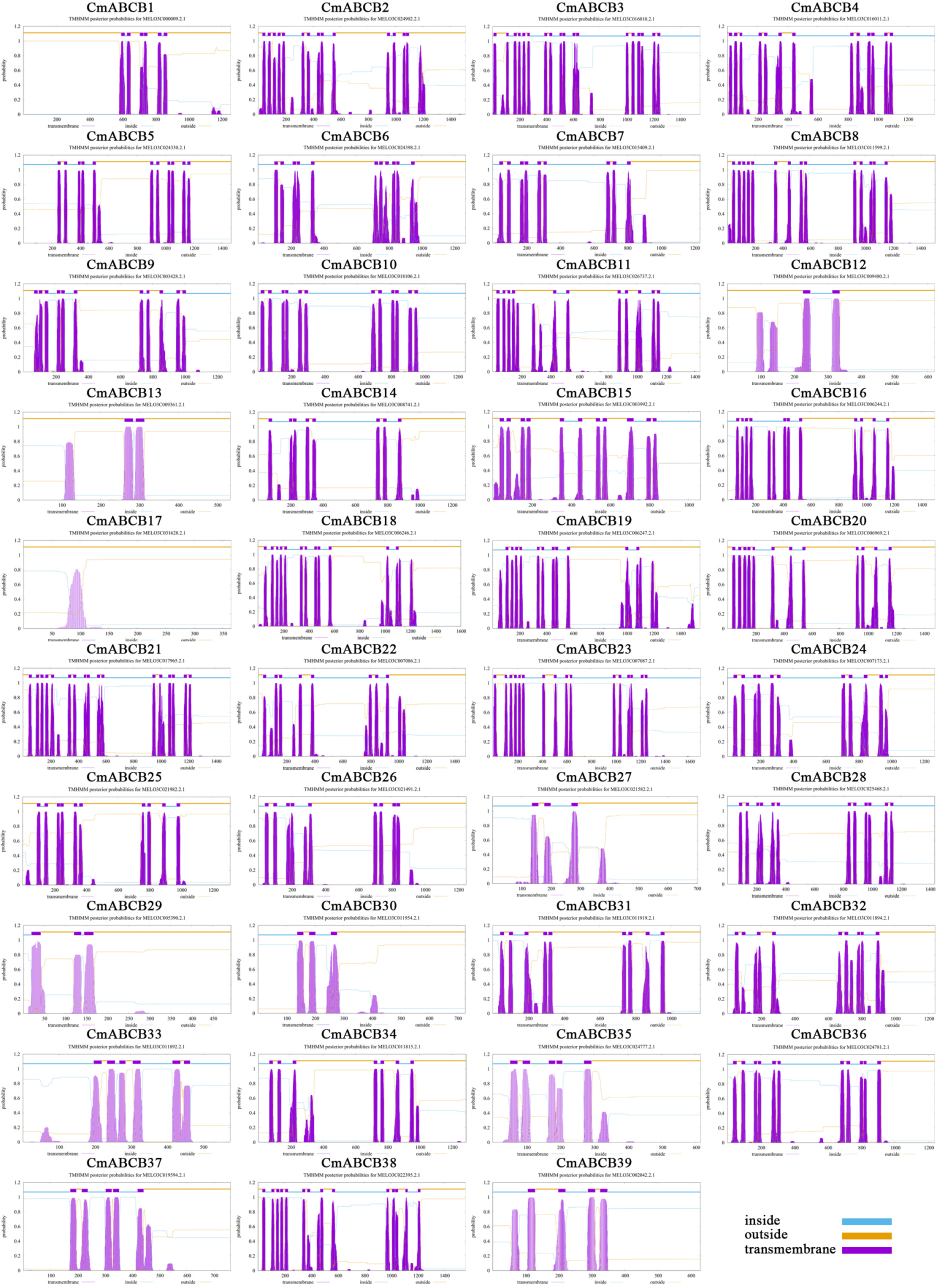
Predicted transmembrane regions of CmABCB proteins. The purple peaks on the top represent the predicted transmembrane helices.

Through comprehensive bioinformatic analyses, we identified 39 *ABCB* genes in melon, with detailed information provided in **Table 1**. The CmABCB proteins ranged in length from 356 (CmABCB17) to 1,690 (CmABCB23) amino acids, with predicted molecular weights (MW) of 42.15–189.01 kDa. The theoretical isoelectric points (pI) values ranged from 5.78 to 9.57. The number of exons in *CmABCB* genes ranged from 3 to 35. Online prediction of subcellular localization indicated that *CmABCB1* was localized to mitochondria, while *CmABCB8*, *CmABCB15*, *CmABCB22*, and *CmABCB23* were localized to vacuoles. Most remaining CmABCB family members were predicted to localize to the plasma membrane, while *CmABCB4*, *CmABCB6*, *CmABCB12*, and *CmABCB20* exhibited dual localization to the plasma membrane and cytoplasm. Transmembrane domain prediction revealed that all CmABCB proteins, except *CmABCB17*, contained varying numbers of transmembrane regions, as shown in **Figure 1**. Three-dimensional structural prediction revealed that CmABCB8, CmABCB11, CmABCB14, CmABCB20, CmABCB21, CmABCB22, and CmABCB23 share relatively high structural similarity (**Supplementary Figure S3**). In addition, SSR analysis identified 141 SSRs across 29 *CmABCB* genes, with mono−nucleotide repeats being the most abundant (**Supplementary Table S3**).

**Table 1:** Information of *CmABCB* genes and properties of the deduced proteins.

Gene name	Locus ID	Exon number	ORF(bp)	Length(aa)	pI	Mol wt(Da)	Predicted subcellular localization
*CmABCB1*	MELO3C000009.2.1	8	3771	1256	9.57	139809.90	Mitochondrion
*CmABCB2*	MELO3C024902.2.1	10	4539	1512	6.14	168440.31	Cell membrane
*CmABCB3*	MELO3C016010.2.1	13	4653	1550	6.47	173601.60	Cell membrane
*CmABCB4*	MELO3C016011.2.1	11	4146	1381	6.83	154862.60	Cell membrane. Cytoplasm
*CmABCB5*	MELO3C024330.2.1	9	4398	1465	8.59	160295.94	Cell membrane
*CmABCB6*	MELO3C024398.2.1	11	3816	1271	5.78	141792.18	Cell membrane. Cytoplasm
*CmABCB7*	MELO3C015409.2.1	9	3705	1234	9.18	134810.85	Cell membrane
*CmABCB8*	MELO3C011599.2.1	26	4527	1508	7.75	169225.70	Vacuole
*CmABCB9*	MELO3C003428.2.1	12	3861	1286	8.48	142119.24	Cell membrane
*CmABCB10*	MELO3C018106.2.1	7	3750	1249	8.68	137841.93	Cell membrane
*CmABCB11*	MELO3C026737.2.1	35	4317	1438	6.32	162163.77	Cell membrane
*CmABCB12*	MELO3C009480.2.1	10	1869	622	8.71	69372.15	Cell membrane. Cytoplasm
*CmABCB13*	MELO3C009361.2.1	7	1605	534	5.82	58640.09	Cell membrane
*CmABCB14*	MELO3C008741.2.1	13	3858	1285	8.73	138911.81	Cell membrane
*CmABCB15*	MELO3C003992.2.1	20	3186	1062	9.27	120735.17	Vacuole
*CmABCB16*	MELO3C006244.2.1	11	4497	1498	8.71	167826.21	Cell membrane
*CmABCB17*	MELO3C031628.2.1	9	1098	365	9.30	42151.62	Cell membrane
*CmABCB18*	MELO3C006246.2.1	12	4806	1601	8.33	179764.72	Cell membrane
*CmABCB19*	MELO3C006247.2.1	12	4650	1549	8.15	173664.95	Cell membrane
*CmABCB20*	MELO3C006969.2.1	11	4440	1479	8.33	164260.94	Cell membrane. Cytoplasm
*CmABCB21*	MELO3C017965.2.1	11	4521	1506	7.75	168371.05	Cell membrane
*CmABCB22*	MELO3C007086.2.1	27	4452	1483	6.27	165756.75	Vacuole
*CmABCB23*	MELO3C007087.2.1	29	5073	1690	7.23	189010.78	Vacuole
*CmABCB24*	MELO3C007173.2.1	12	3804	1267	7.56	136656.24	Cell membrane
*CmABCB25*	MELO3C021982.2.1	11	3930	1309	8.64	142993.39	Cell membrane
*CmABCB26*	MELO3C021491.2.1	8	3747	1248	8.61	135332.76	Cell membrane
*CmABCB27*	MELO3C021582.2.1	18	2112	703	8.48	78929.42	Cell membrane
*CmABCB28*	MELO3C025468.2.1	12	4302	1433	6.45	159608.97	Cell membrane
*CmABCB29*	MELO3C005390.2.1	13	1359	493	7.19	53665.65	Cell membrane
*CmABCB30*	MELO3C011954.2.1	18	2184	727	7.26	80910.42	Cell membrane
*CmABCB31*	MELO3C011919.2.1	7	3495	1164	8.82	128172.10	Cell membrane
*CmABCB32*	MELO3C011894.2.1	10	3723	1240	8.64	139204.12	Cell membrane
*CmABCB33*	MELO3C011892.2.1	10	1728	575	8.99	65329.20	Cell membrane
*CmABCB34*	MELO3C011815.2.1	12	3864	1287	8.70	139318.41	Cell membrane
*CmABCB35*	MELO3C024777.2.1	3	1845	614	8.81	68724.75	Cell membrane
*CmABCB36*	MELO3C024781.2.1	10	3720	1239	6.45	136596.68	Cell membrane
*CmABCB37*	MELO3C019594.2.1	20	2289	762	9.25	83663.72	Cell membrane
*CmABCB38*	MELO3C022395.2.1	12	4686	1561	7.96	174448.46	Cell membrane
*CmABCB39*	MELO3C002042.2.1	17	1890	629	9.06	67668.09	Cell membrane

ORF, pI, and Mol wt represent open reading frame, isoelectric point, and molecular weight, respectively.

### 3.2 Phylogenetic analysis of the ABCB gene family in melon

**Figure 2:**
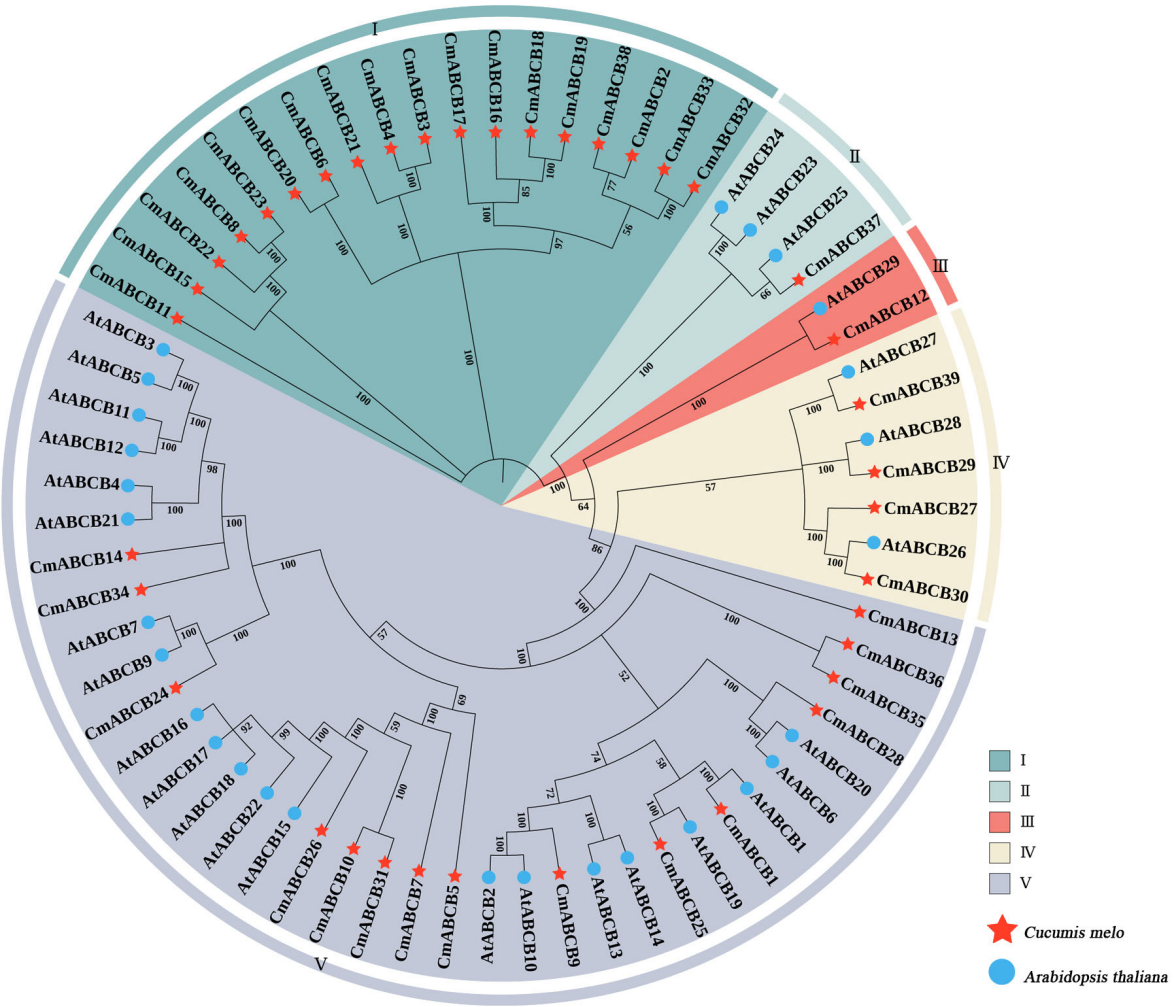
Phylogenetic analysis of *CmABCBs* and *AtABCBs.* Members belonging to different subgroups are shown in different colors. Red pentagram indicates *C. melo*; Blue circle indicates *A. thaliana*.

To investigate the potential functions of ABCB gene family in melon, we constructed a phylogenetic tree using ABCB protein sequences from *C. melo* and *A. thaliana*. The combined ABCB proteins were clustered into five distinct groups, designated Groups I-V (**Figure 2**). Seven orthologous gene pairs were identified between melon and *A. thaliana*, with Groups II, III, IV, and V containing 1, 1, 3, and 2 pairs, respectively. This suggests that these orthologous genes existed prior to the speciation of melon and *A. thaliana* and may remain conserved biological functions. Notably, Group I contained only *CmABCB* genes, forming a distinct melon-specific clade. Additionally, eight paralogous gene pairs were identified in melon, with six pairs in Group I and two pairs in Group V. This indicates that these paralogous *CmABCB* genes have undergone melon-specific expansion, which may be associated with their unique physiological adaptations.

### 3.3 Analysis of Conserved motifs, domains, and gene structures in the melon ABCB gene family

**Figure 3:**
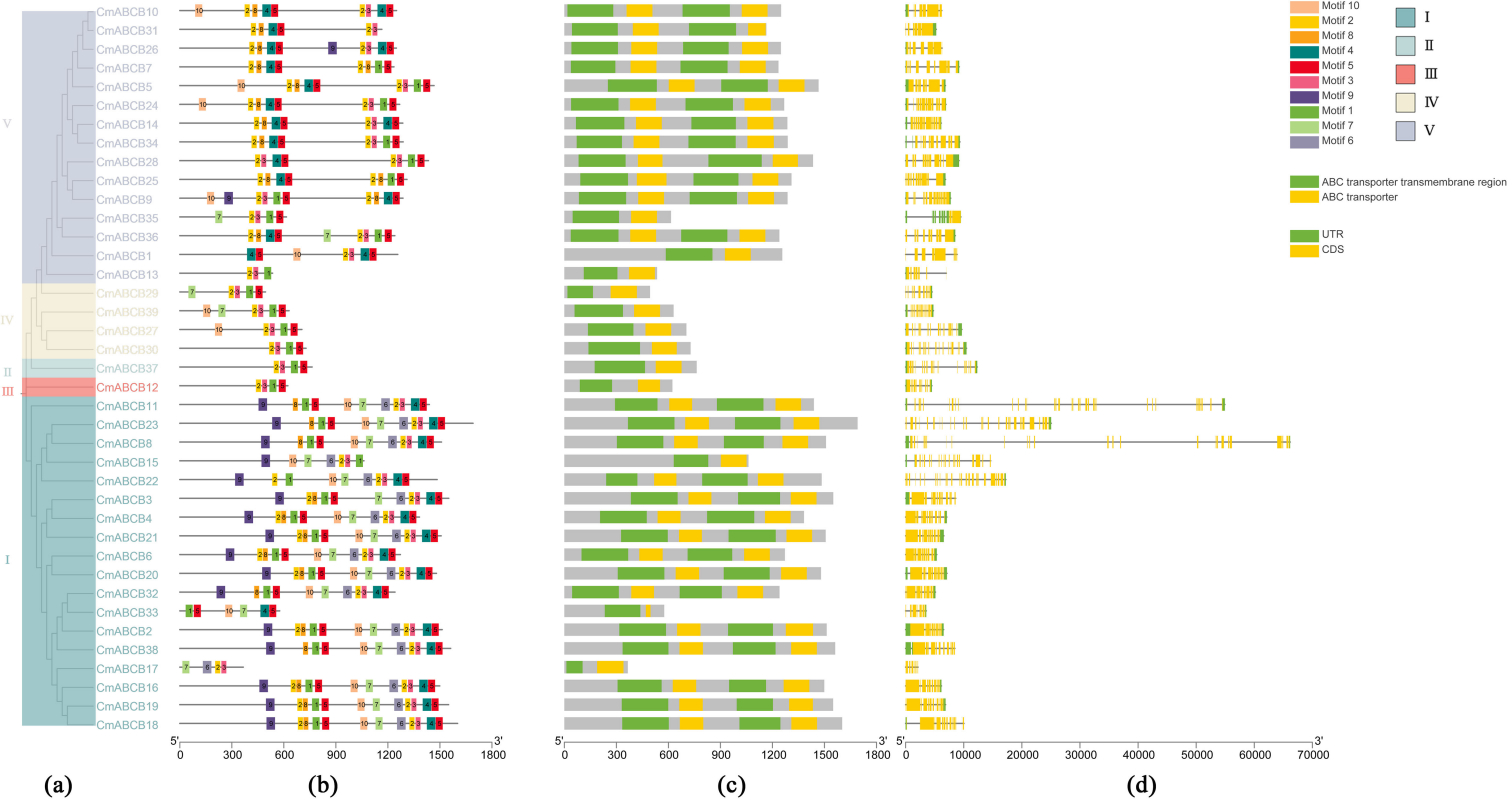
Conserved structure of CmABCB protein family. **(a)** Phylogenetic tree of CmABCB proteins, Members belonging to different subgroups are shown in different colors. **(b)** Motif pattern of CmABCB proteins, where the ten different colored rectangles represent the ten motifs. **(c)** *CmABCB* gene conserved domain. The ABC transporter transmembrane regions are shown by green squares, and ABC transporter domains are shown by yellow squares. **(d)** Intron and exon structure of *CmABCB* genes, where green indicates UTR and yellow indicates CDS.

By integrating phylogenetic analysis, conserved motif identification, domain annotation and gene structure analysis, the evolutionary relationships within the CmABCB gene family were further clarified (**Figure 3**). The results showed that CmABCB family members clustered in the same evolutionary branch typically shared similar conserved motif compositions and gene structures. Conserved domain annotation via NCBI CDD and SMART revealed that members of Groups II, III, and IV harbored one transmembrane domain (TMD) and one nucleotide-binding domain (NBD), while most members of Groups I and V contained two TMDs and two NBDs—consistent with the classification of half-size and full-size ABCB transporters. Consistent with the gene structure analysis, the number of exons in *CmABCBs* ranged from 3 to 35, reflecting significant structural diversity within the family. Notably, *CmABCB8* and *CmABCB11* were predicted to contain long introns exceeding 40,000 bp, which may affect their transcriptional regulation.

### 3.4 Chromosomal localization and collinearity analysis of the melon ABCB gene family

**Figure 4:**
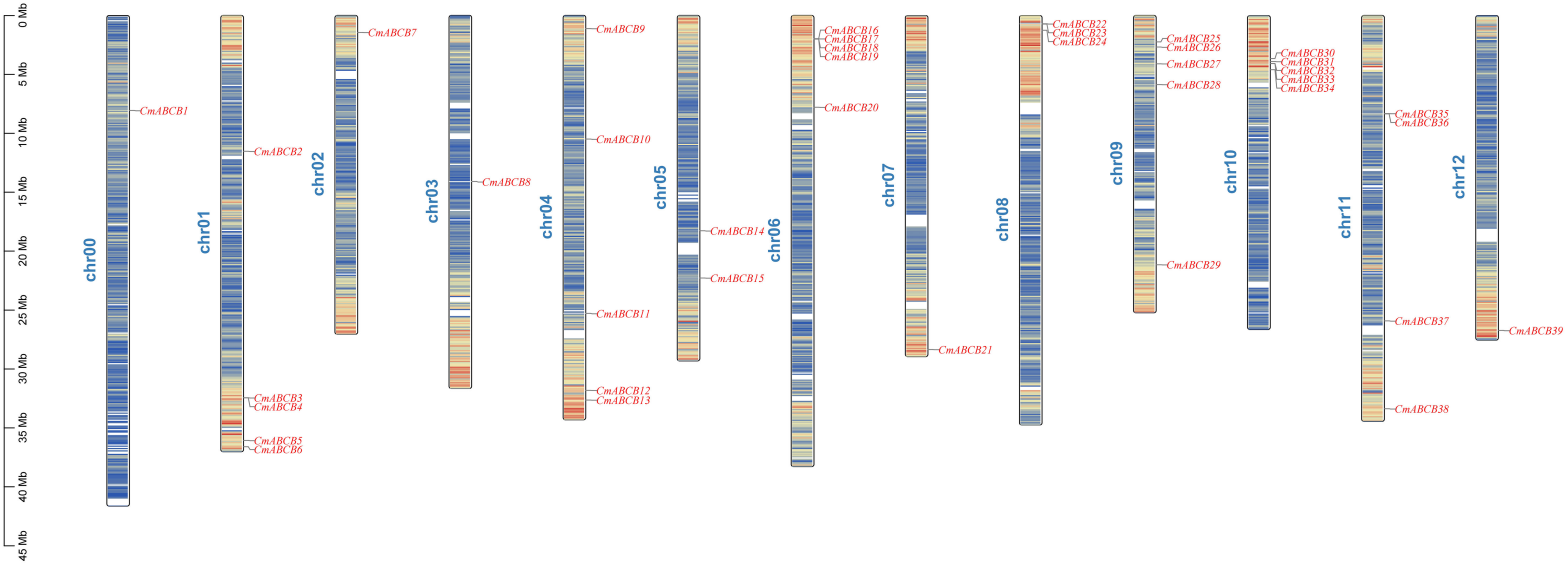
Chromosomal location of *CmABCB* genes. *CmABCB1* is positioned on an unanchored scaffold (chr00). A color gradient from blue (low density) to red (high density) depicts gene density across chromosomal segments. The vertical axis shows chromosome length (Mb).

**Figure 5:**
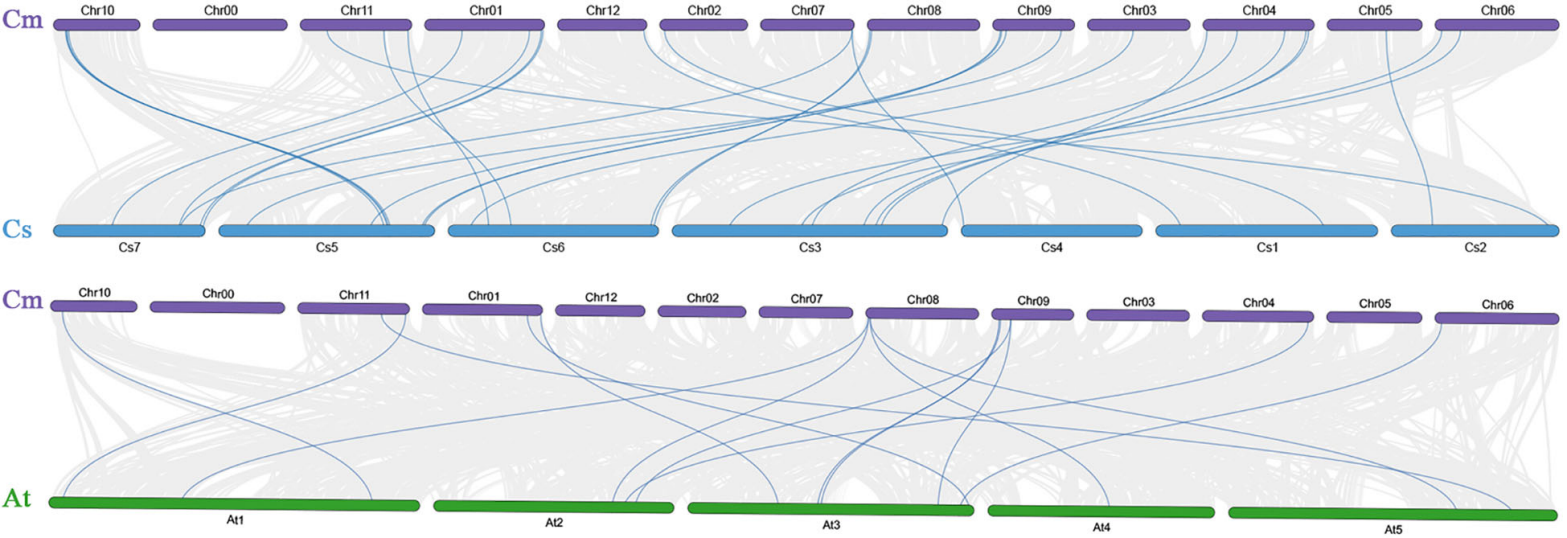
*CmABCB* collinearity analysis. Cm, *C. melo*; Cs, *C. sativus*; At, *A. thaliana.* The blue lines indicate collinear gene pairs.

We performed chromosomal localization analysis to map the 39 *CmABCB* genes to the melon genome, and they were named *CmABCB1* to *CmABCB39* according to their physical order from the short arm to the long arm of each chromosome (**Figure 4**). Except for *CmABCB1*, which was positioned on an unanchored scaffold (chr00), the remaining 38 genes were unevenly distributed across all 12 chromosomes. Chromosomes 1 to 12 harbored 5, 1, 1, 5, 2, 5, 1, 3, 5, 5, 4, and 1 *CmABCB* gene(s), respectively, with most genes concentrated in telomeric regions, suggesting potential clustering of functional homologous genes. To investigate the evolutionary relationships of the CmABCB family members, collinearity analysis was performed on the genomes of *C. melo*, *C. sativus*, and *A. thaliana* (**Figure 5**). The melon ABCB gene family exhibited specific gene loss and expansion events. A total of 30 and 15 collinear gene pairs were identified between *C. melo* and *C. sativus* and between *C. melo* and *A. thaliana*, respectively (collinear gene pairs are provided in **Supplementary Table S2**). The correspondence between *C. melo* and *C. sativus* was highly conserved, predominantly following a one-to-one relationship. Three *CmABCB* genes showed one-to-two correspondence with *A. thaliana* genes, indicating their important roles in the evolution of the CmABCB gene family. Furthermore, *10 CmABCB* genes showed no collinearity with *CsABCBs*, and 27 *CmABCB* genes lacked collinearity with *AtABCBs*.

### 3.5 GO and KEGG analysis of *CmABCB* genes

**Figure 6:**
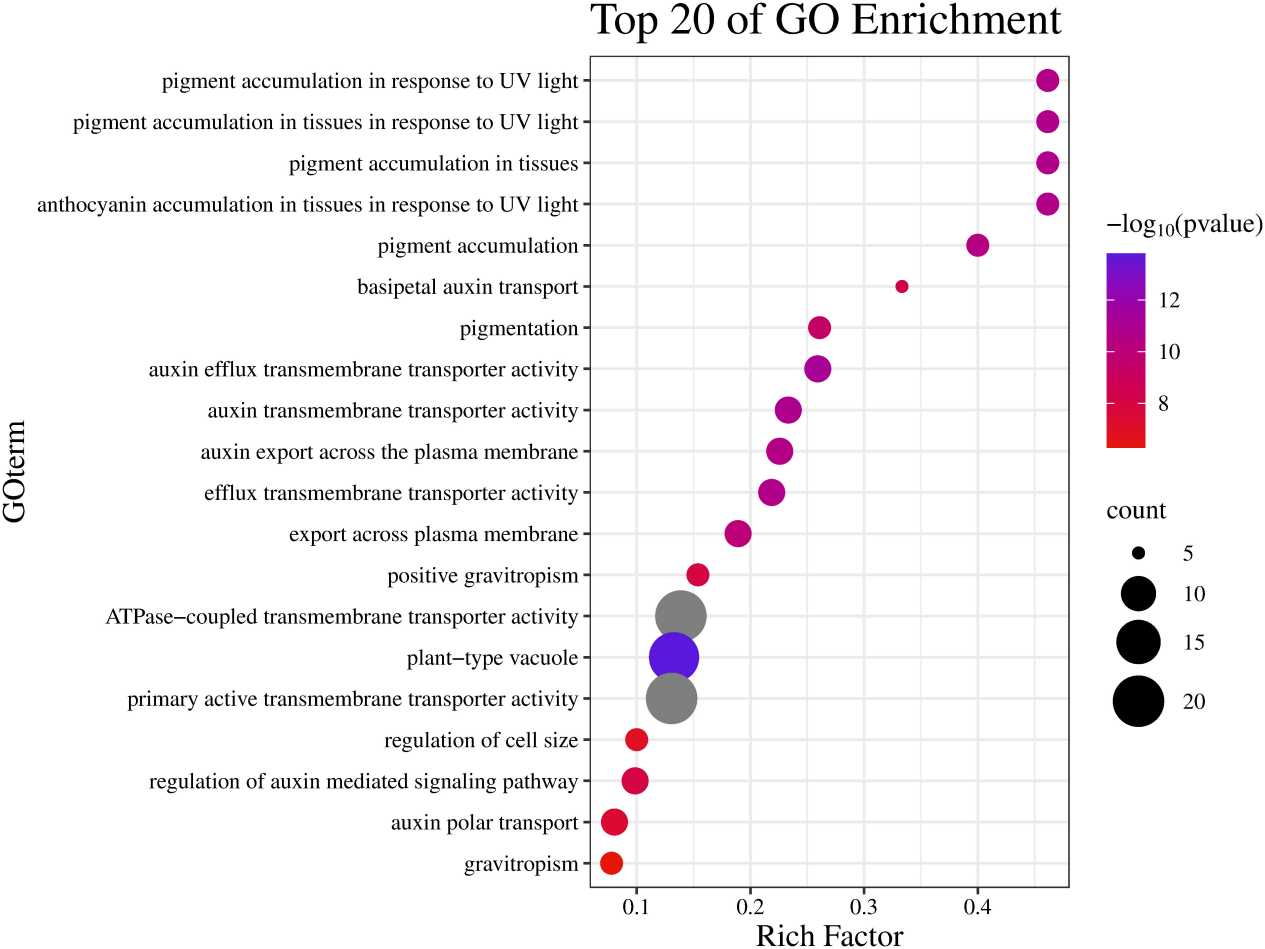
GO enrichment analysis of the *CmABCB* genes. The ordinate is the main GO classification categories, and the abscissa is the enrichment factor.

**Figure 7:**
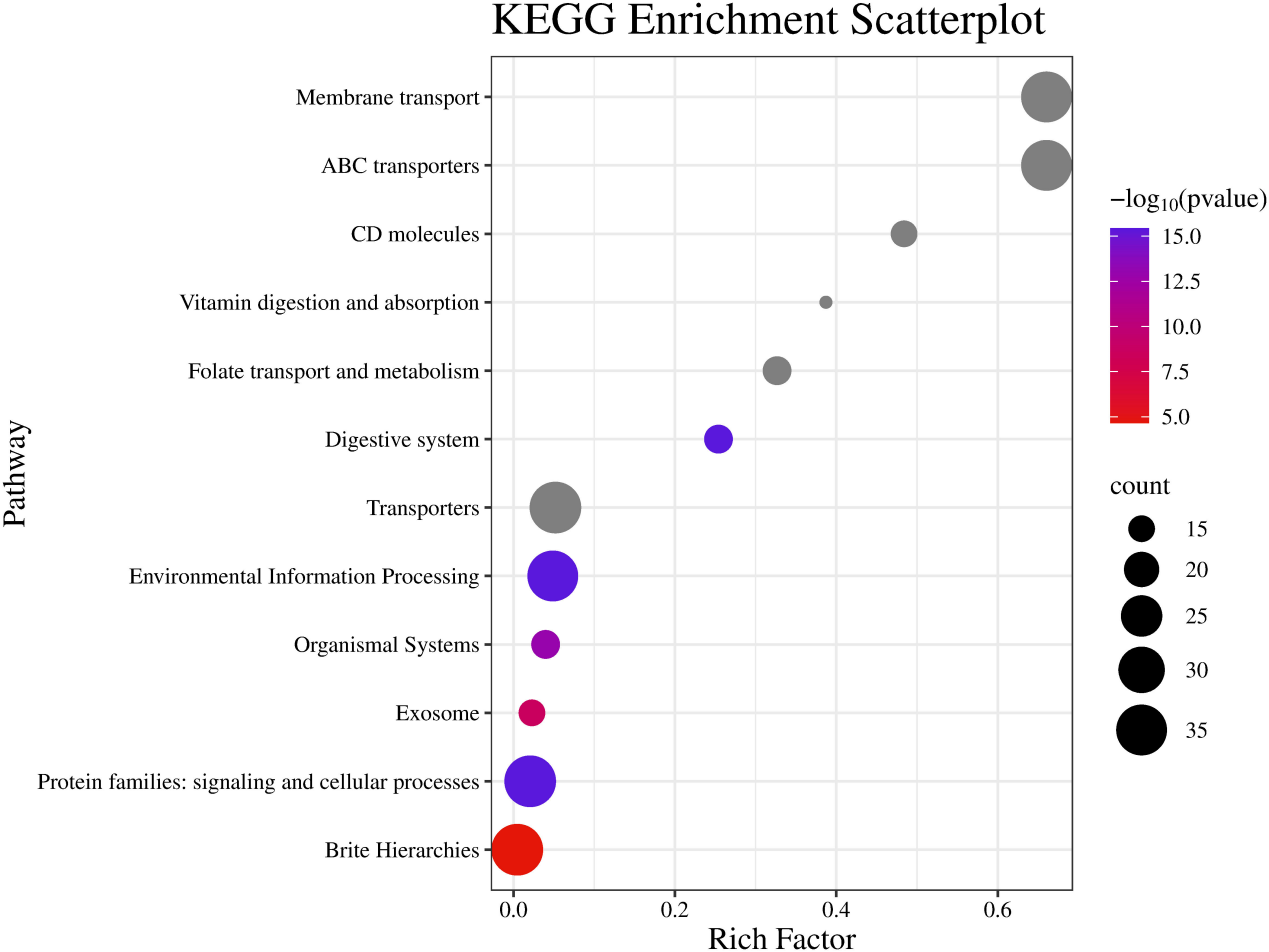
KEGG pathway enrichment analysis of the *CmABCB* genes. The ordinate is the main KEGG classification categories, and the abscissa is the enrichment factor.

GO and KEGG enrichment analyses were performed to clarify the biological functions of *CmABCB* genes. The GO results showed that these genes were significantly enriched in auxin transmembrane transporter activity, auxin efflux transmembrane transporter activity, auxin polar transport, and ATPase-coupled transmembrane transporter activity, which are highly consistent with the typical functional characteristics of ABCB transporters (**Figure 6**). KEGG pathway analysis revealed that the most significantly enriched pathway was ABC transporters, showing the highest enrichment factor and the largest number of enriched genes. The genes were also enriched in membrane transport and related functional pathways (**Figure 7**). These results support that *CmABCB* genes play core roles in transmembrane transport and auxin transport/regulation in melon.

### 3.6 Promoter cis-acting element analysis of the melon ABCB gene family

**Figure 8:**
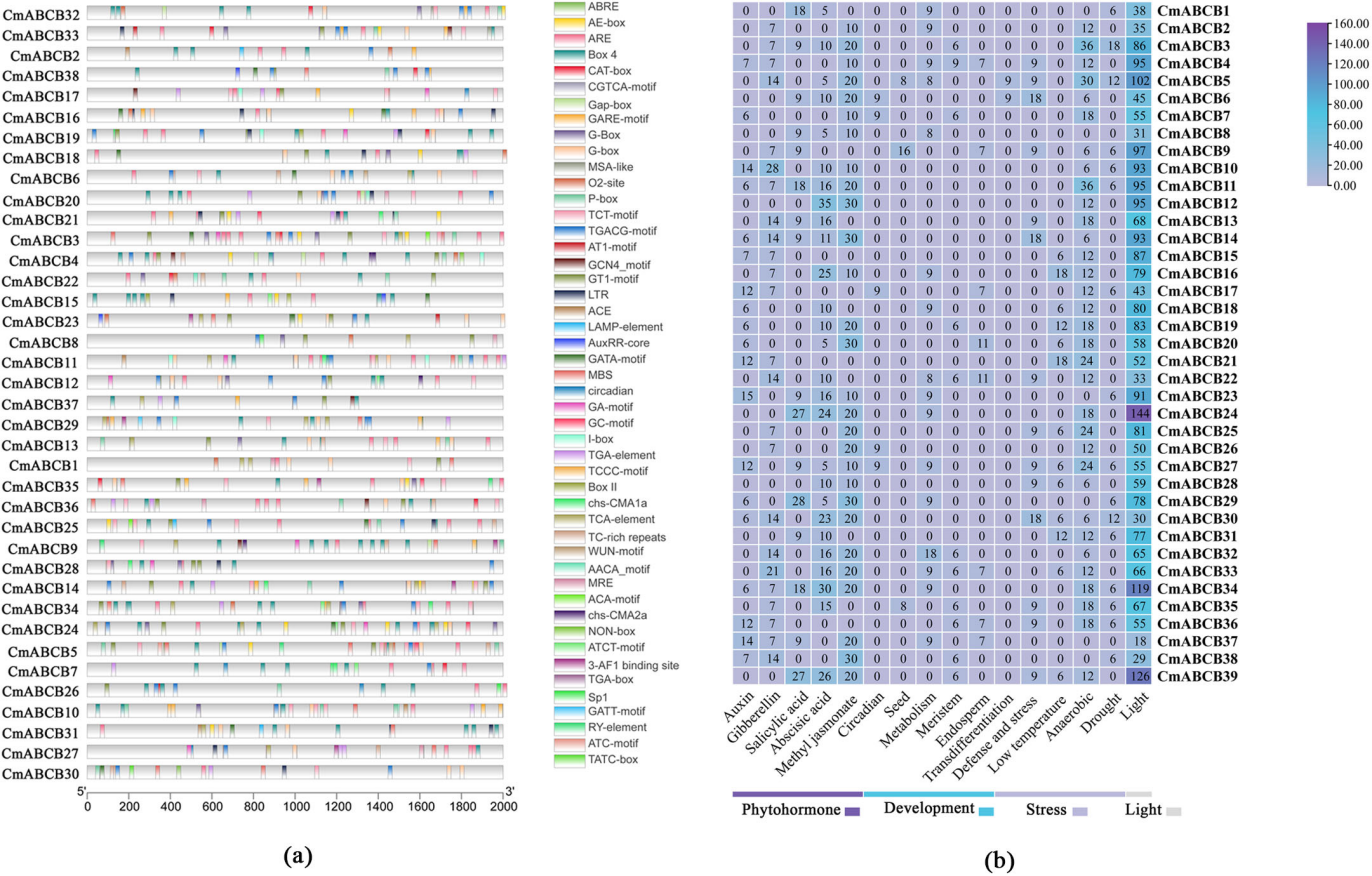
Analysis of *cis*-acting elements in the promoters of *CmABCB* genes. **(a)** Analysis and visualization of *cis*-acting elements in the promoter regions of *CmABCB* genes. **(b)** Composition and counts of *cis*-acting elements in *CmABCB* gene promoters. *Cis*-acting elements are categorized by colored horizontal lines: dark purple for phytohormone, blue for development, light purple for stress, and gray for light responsiveness. The numerical values within the boxes indicate the count of elements for each gene.

We extracted the 2,000 bp promoter regions (upstream of the start codon) of all *CmABCB* genes from the latest melon genome, and identified 47 types of functional *cis*-acting elements (**Figure 8**). These elements were mainly categorized into three groups: plant growth and development, hormone regulation, and abiotic stress tolerance. Among the *cis*-acting elements associated with plant growth and development, light-responsive, circadian rhythms, and seeds development were identified with light-responsive being most prominent. Light-responsive elements (e.g., ACE, AE-box, Sp1) were the most abundant, accounting for approximately 50% of the total. Among the hormone-related *cis*-acting elements, auxin response elements (e.g., AuxRR-core, TGA-element), gibberellin response elements (e.g., GARE-motif, P-box), salicylic acid response elements (TCA-element), abscisic acid response elements (ABRE), and methyl jasmonate response elements (CGTCA-motif, TGACG-motif) were identified. The number of hormone-responsive *cis*-acting elements was second only to those involved in growth and development, with methyl jasmonate and abscisic acid response elements being the most numerous and widely distributed, present in the promoters of almost all *CmABCB* genes. Additionally, abiotic stress-responsive *cis*-acting elements were identified, including wound-responsive element (WUN-motif), low-temperature response element (LTR), and drought response element (MBS), representing the smallest proportion of elements.

### 3.7 Expression profiling of *CmABCB* genes in axillary buds during bud induction and qRT-PCR validation

**Figure 9:**
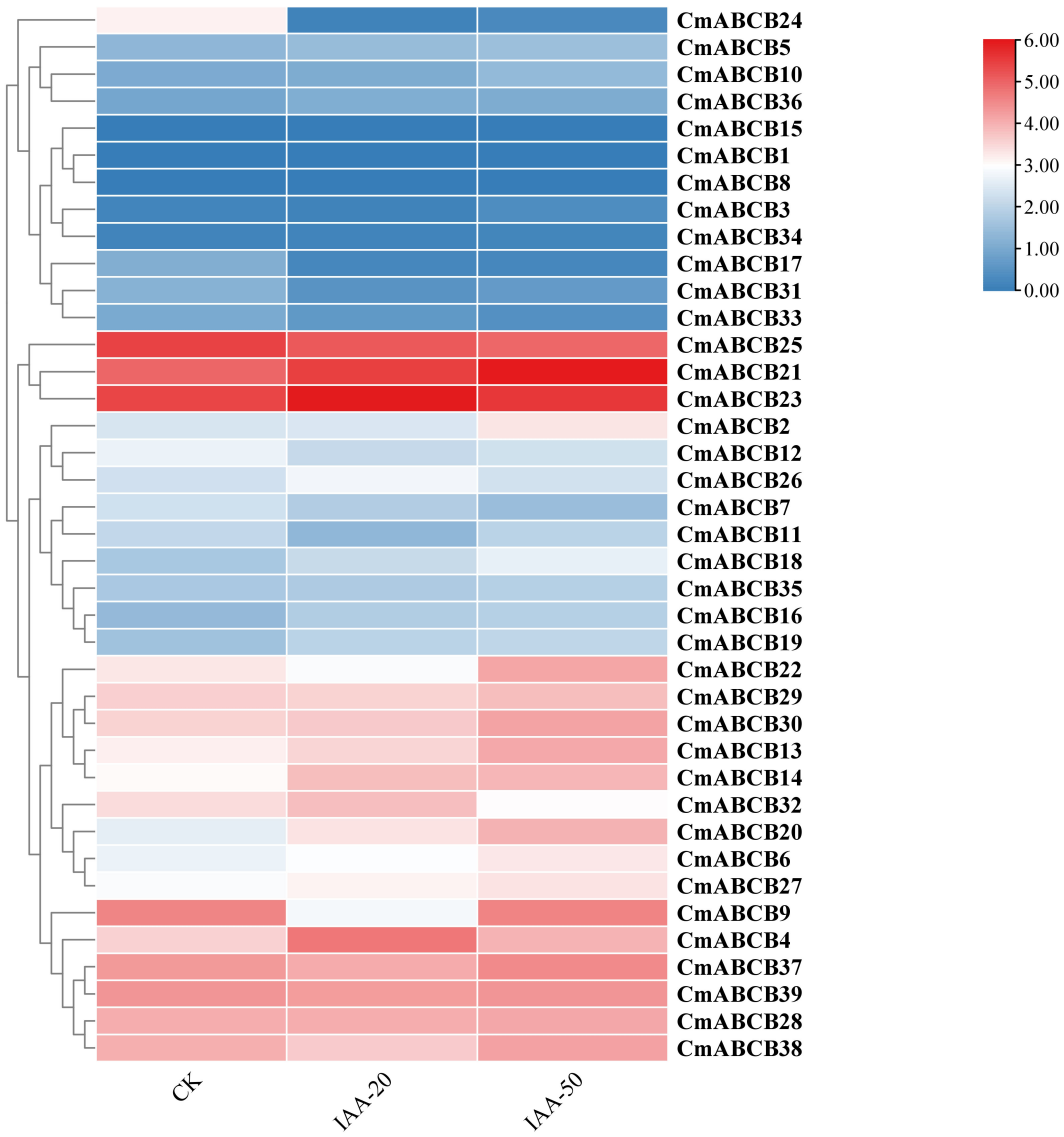
Expression patterns of *CmABCB* genes in axillary buds in response to IAA treatments, with a gradient from blue to red indicating increasing expression levels from low to high.

**Figure 10:**
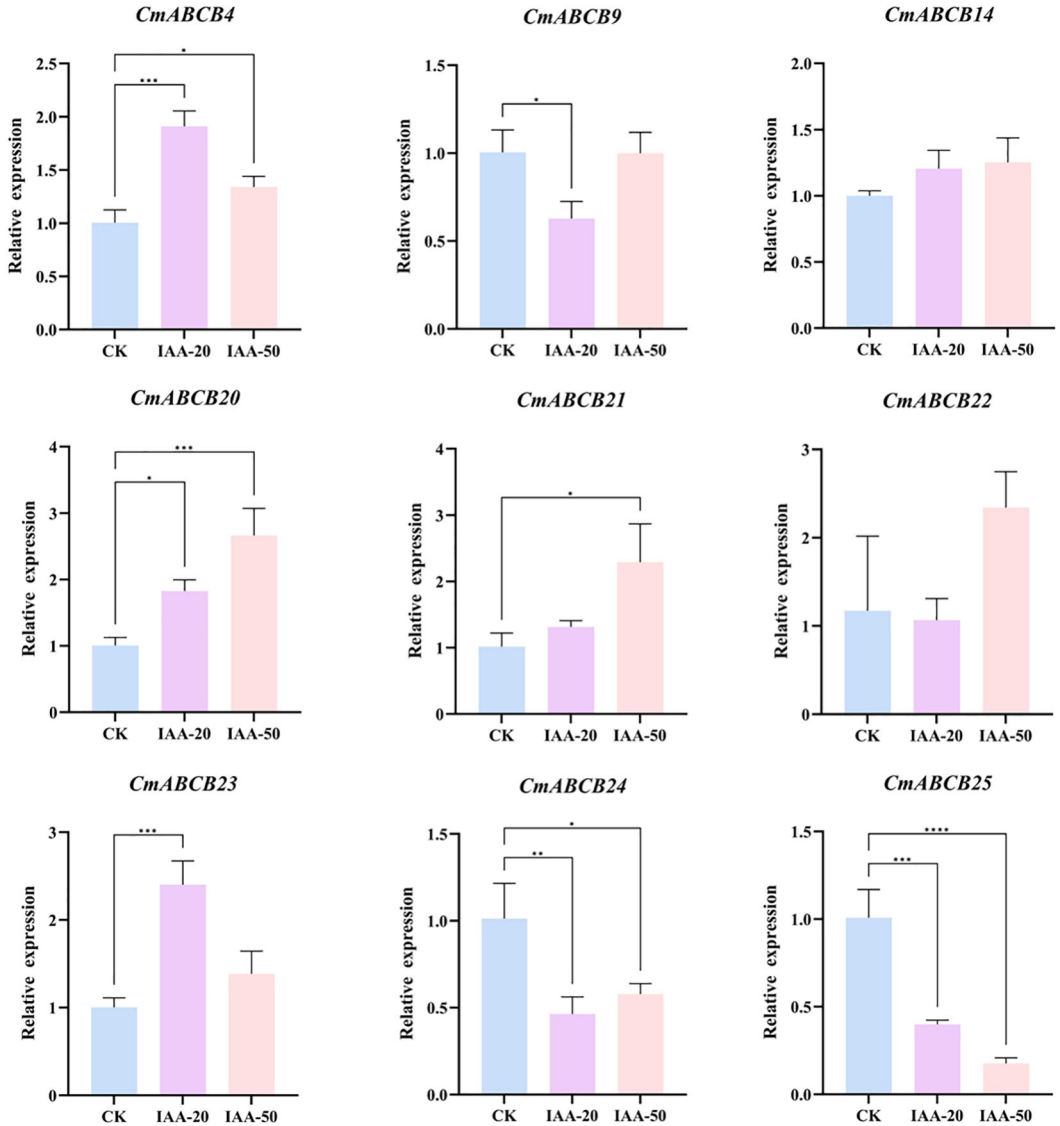
The qPCR results of 9 *CmABCB* genes under different IAA treatments. Different colors represent relative transcript levels on different treatments. Asterisks indicate significance of differences between groups (one-way ANOVA). *p < 0.05; **p < 0.01; ***p < 0.001; ****p < 0.0001.

To further investigate the potential roles of *CmABCB* genes in IAA mediated axillary bud development, we performed RNA sequencing (RNA-seq) on melon axillary buds treated with three IAA concentrations (0 mg/L, 20 mg/L and 50 mg/L). A heatmap was generated based on the gene expression data, showing the expression patterns of *CmABCBs* under different IAA treatments (**Figure 9**). The expression levels of *CmABCB* genes varied substantially. *CmABCB1*, *CmABCB8*, and *CmABCB15* showed relatively low expression levels, whereas *CmABCB21*, *CmABCB23*, and *CmABCB25* exhibited high expression levels, suggesting their potential importance during axillary bud development. Comparison of *CmABCB* expression levels across different IAA treatments showed that most genes did not exhibit significant changes in expression. However, distinct expression patterns were observed for several genes: *CmABCB14*, *CmABCB20*, and *CmABCB21* showed sustained upregulation; *CmABCB4* and *CmABCB23* displayed an initial increase followed by decreased expression; *CmABCB25* demonstrated consistent downregulation; and *CmABCB9*, *CmABCB22*, and *CmABCB24* exhibited an initial decrease followed by increased expression. These results indicate that these genes are closely associated with axillary bud growth in melon. Based on the transcriptome analysis, we selected nine *CmABCB* genes with significant expression differences for validation by qRT-PCR. The results showed that the expression trends of these *CmABCB* genes under different IAA treatments were highly consistent with the RNA-seq data (**Figure 10**), confirming the reliability of the transcriptome data in this study and providing a solid foundation for further investigation of *CmABCB* gene functions in axillary bud development.

## 4 Discussion

Shoot branching initiates with the formation of axillary buds, which differentiate into axillary buds that can either undergo outgrowth into vegetative stems, inflorescences, or even flowering branches. This process constitutes a core survival strategy, allowing plants to mitigate shading stress through architectural modification, thereby optimizing light capture and adaptation to nutrient-limited environments (Evers et al., 2011).From an agricultural and horticultural perspective, branching is also a critical agronomic trait. Different crops have evolved unique branching patterns to balance vegetative and reproductive growth, which is essential for maintaining crop product quality and productivity (Smith and Samach, 2013; Xiong et al., 2024). For graminaceous crops such as rice (Zhang et al., 2024) and wheat (Ren et al., 2024), promoting tillering to increase the number of effective panicles is an effective approach to improve yield. In fruit tree cultivation, proper pruning and training can optimize canopy structure, improve light distribution, and direct nutrient transport, thereby promoting flower bud differentiation and significantly improving fruit yield and quality (Zhang et al., 2023). Melon displays vigorous branching vigor; excessive branching induces intense competition for photosynthates, which not only reduces nutrient supply to individual fruits (negatively affecting fruit size and sugar accumulation) but also causes field shading and elevates the risk of pest and diseases infestations (Singh et al., 2022). Thus, regulating branching intensity is essential for ensuring melon yield and quality, a practice that inevitably elevates cultivation labor costs. Currently, the genetic basis and molecular regulatory mechanisms underlying melon branching remain poorly characterized. This knowledge gap represents a major bottleneck for developing melon varieties with ideal plant architecture and achieving cost-effective production through genetic improvement. Following axillary bud formation, its fate, which is either continuing to grow and develop into branches or maintaining dormancy, is regulated by the interaction of genetic factors, plant hormones, environmental cues, and nutrients (Domagalska and Leyser, 2011). Previous studies have established that auxin levels are a key determinant regulating axillary bud activation or dormancy (Thimann and Skoog, 1933). Given that ABCB proteins serve as key auxin transporters in various plant species (Xu et al., 2014), whether they are involved in the regulating melon axillary bud development warrants systematic investigation, this is the core focus of the present study.

In the present study, we performed the first systematic genome-wide identification of the ABCB gene family in melon and identified a total of 39 *CmABCB* genes. The number of ABCB genes (39) is higher than that in *A. thaliana* (28), *Oryza sativa* (27) and *Zea mays* (31), and is consistent with that in soybean (Pang et al., 2013; Zou et al., 2025). The variation in ABCB gene family size across species is likely attributed to the dynamic interplay of gene duplication and loss events that the gene family has undergone during evolutionary divergence (Marchant et al., 2021). Our analysis revealed that CmABCB proteins are diverse in amino acid length, isoelectric point (pI), and predicted molecular weight, with an average molecular weight of 131.14 kDa and an average pI of 7.98 which are comparable to those of ABCB proteins in other crops. The polymorphism in amino acid sequence length and variations in protein domain composition fully reflect the evolutionary divergence within the CmABCB gene family. However, despite such sequence and structural divergence, the retention and duplication patterns of the ABCB gene family across species are subject to similar evolutionary constraints, and its members remain highly conserved in their core biological functions (Airoldi and Davies, 2012). Subcellular localization prediction of the 39 CmABCB proteins revealed that they were localized to the cell membrane, cytoplasm, mitochondria, and vacuoles. Notably, the majority of *CmABCB* genes (34/39, 87.18%) were predicted to localize to the plasma membrane, indicating that the melon ABCB gene family plays a crucial role in mediating intercellular auxin transport.

Phylogenetic analysis revealed that these *CmABCB* genes were clustered into five distinct groups (I-V), with Group V harboring the largest number of members (18), while Groups II and III contained only one member each. Notably, Group I was composed solely of melon-specific members, forming a distinct species-specific clade, which reflects species-specific expansion of the *CmABCB* genes in melon, which may be linked with its unique shoot branching regulatory mechanisms or other species-specific agronomic traits (Chen et al., 2024). The *CmABCB* genes exhibited high homology with *AtABCB* genes from *A.thaliana*, particularly in Group V, suggesting conserved biological functions throughout evolution. Chromosomal localization and synteny analysis demonstrated that the 39 *CmABCB* genes were randomly distributed across all melon chromosomes, with higher gene densities on Chr01, Chr06, Chr09, and Chr10 (collectively harboring 20/39, 51.28% of all *CmABCB* genes). This non-random distribution pattern may be associated with local gene duplication events in the melon genome. We detected tandem duplication events within the CmABCB gene family. Segmental duplications frequently occurring in genomic evolution and often associated with whole-genome duplication events may enable duplicated genes to acquire novel functions, thereby enhancing plant adaptation to various environmental stresses (Cannon et al., 2004). Furthermore, collinearity analysis revealed significantly more syntenic gene pairs between *C. melo* and *Cucumis sativus* than between melon and *A. thaliana*. This synteny pattern is consistent with the phylogenetic relationships, as closely related species within the Cucurbitaceae family, melon and cucumber maintain high genomic synteny, whereas *A. thaliana*, a member of the early-diverged Brassicaceae family with a distant phylogenetic relationship, has undergone more extensive lineage-specific genomic rearrangements, resulting in lower retention of homologous gene pairs.

Although CmABCB proteins exhibit substantial diversity in physicochemical properties, the conserved motif composition of these proteins and the gene structures of their corresponding *CmABCB* genes are highly conserved. The 39 identified *CmABCB* genes exhibit extensive variation in exon-intron organization with exon numbers ranging from 3 to 35 and some members harboring ultra-long introns. Previous studies have demonstrated that an increase in intron number is usually accompanied by the extension of gene sequences and may enhance recombination frequency among genes, thereby promoting functional divergence of the gene family (Shabalina et al., 2010). Analysis of conserved motifs in CmABCB proteins identified 10 distinct motifs, among which Motif 2 (present in all members except CmABCB33) and Motif 5 (detected in 82.05% of members) were present in most CmABCB proteins, suggesting that these are highly conserved core functional units of the family. Combined analysis of conserved motifs and gene structures of *CmABCB* members revealed that members clustered in the same phylogenetic clade share similar conserved motif compositions and gene structures, while significant differences were observed among members of different clades. This indicates that members within the same clade exhibit conservation and similar functions, which is consistent with the findings of Wang et al (Wang et al., 2025) and Yu et al (Yu et al., 2017). In addition, seven CmABCB proteins (CmABCB8, CmABCB11, CmABCB14, CmABCB20, CmABCB21, CmABCB22, CmABCB23) exhibited particularly high three-dimensional structural similarity. SSR analysis identified 141 simple sequence repeats across 29 *CmABCB* genes, providing a resource for future molecular marker development.

GO and KEGG enrichment analyses confirmed that the *CmABCB* genes are significantly enriched in ATP binding, auxin efflux, plasma membrane localization, and the ABC transporter pathway, which fully aligns with their core roles in transmembrane transport and auxin regulation. To further explore the transcriptional regulatory mechanisms underlying these functions, we analyzed the *cis*-acting elements in their promoter regions. They contain multiple *cis*-acting elements that not only provide specific binding sites for transcriptional regulatory proteins but also directly regulate tissue-specific gene expression and response to external environmental signals (Hernandez-Garcia and Finer, 2014; Shen and Li, 2023). Based on the promoter sequences of the 39 *CmABCB* genes, we predicted their *cis*-acting elements and found that the promoter regions harbor various response elements associated with light, plant hormones, low temperature, drought, and other stimuli, indicating that *CmABCB* genes may play roles in these responses. Notably, 19 *CmABCB* genes contained auxin-related *cis*-acting elements (e.g., AuxRR-core, TGA-element), suggesting that these genes are directly regulated by auxin signaling and may participate in auxin-mediated axillary bud development in melon. *CmABCB4*, *CmABCB14*, *CmABCB20*, *CmABCB21* and *CmABCB23* contained abundant auxin-responsive *cis*-acting elements, with 15 in *CmABCB23*, 12 in *CmABCB21*, 7 in *CmABCB4*, and 6 in each of *CmABCB14* and *CmABCB20*. Notably, all these *CmABCB* genes exhibited significant expression changes under different concentrations of exogenous IAA treatment. In contrast, *CmABCB1*, *CmABCB3* and *CmABCB8*, which lack auxin-responsive *cis*-acting elements, showed no obvious expression alterations under the same IAA concentrations. These results indicate that the abundant auxin-responsive elements in these *CmABCB* genes endow them with the ability to rapidly respond to IAA signals, potentially modulating auxin transport efficiency in melon axillary buds and thereby regulating bud outgrowth.

By analyzing the expression profiles of *CmABCB* genes in melon axillary buds under different IAA concentrations, we gained insights into their expression dynamics during axillary bud growth. Our results demonstrated that *CmABCB* genes showed heterogeneous expression responses to varying IAA concentrations (Student’s t-test, P<0.05): most genes exhibited no significant expression changes, while a subset of genes displayed distinct and concentration-dependent expression patterns. Specifically, the expression levels of *CmABCB14*, *CmABCB20*, and *CmABCB21* showed a positive correlation with IAA concentration, which is consistent with the functional characteristics of known *AtABCB1* and *AtABCB21* in *A. thaliana* (Chen et al., 2023).These genes likely enhance auxin efflux activity to establish and maintain a local auxin concentration gradient in axillary buds. Consistent with the classic apical dominance model, this gradient may contribute to inhibiting axillary bud outgrowth by maintaining high auxin levels in the bud meristem, thereby playing key roles in axillary bud dormancy regulation. In contrast, *CmABCB4* and *CmABCB23* showed an initial increase followed by a decrease in expression with rising IAA concentration, while *CmABCB25* exhibited continuous downregulation, and *CmABCB9*, *CmABCB22*, and *CmABCB24* displayed a trend of initial decrease then increase. These diverse patterns imply that these genes may participate in fine-tuning auxin signaling at different stages of axillary bud development. Collectively, these findings indicate that *CmABCB* genes play important roles in melon axillary bud development. Furthermore, we conducted qRT-PCR analysis to validate the RNA-seq results, further ensuring the reliability of the expression data.

## 5 Conclusion

This study identified a total of 39 *CmABCB* genes in melon and systematically analyzed their physicochemical properties, phylogenetic relationships, gene structures, synteny, and promoter *cis*-acting elements. Furthermore, analysis of their expression profiles in axillary buds in response to IAA treatment revealed that multiple *CmABCB* genes exhibited differential responses during axillary bud development. Among these differentially expressed genes, the expression levels of *CmABCB14*, *CmABCB20*, and *CmABCB21* showed a significant positive correlation with IAA concentration. Combined with the conserved function of their homologous genes in Arabidopsis, these genes are hypothesized to act as auxin efflux carriers, playing key roles in inhibiting axillary bud outgrowth in melon. Our findings not only fill the knowledge gap in the systematic characterization of the melon ABCB gene family but also provide a key theoretical basis for elucidating the molecular mechanism of IAA-mediated branching regulation in melon. Additionally, these results offer valuable candidate genes and insights for the genetic improvement of ideal plant architecture in melon breeding, and lay a foundation for further functional verification of *CmABCB* genes in axillary bud development.

## Data Availability

The raw sequencing data generated in this study can be found in the SRA (https://www.ncbi.nlm.nih.gov/sra/) of NCBI under accession number PRJNA1451451.

## Supplementary material

Supplementary Figure S1 Bioinformatics workflow for genome-wide identification and basic characterization of the ABCB gene family in melon (*Cucumis melo* L.).

Supplementary Figure S2 Representative phenotypes of melon axillary buds under control (CK) and exogenous IAA treatments at Day 0 and Day 14 (The red arrows in the figure are used to indicate the position of the axillary buds).

Supplementary Figure S3 Predicted three-dimensional structures of the 39 CmABCB proteins.
