# Supplementary figures and images for: Genome-wide identification of the ABCB gene family in melon (Cucumis melo L.) and their expression during axillary bud development
Source: Front Plant Sci. 2026 Apr 22;17:1770152. doi: 10.3389/fpls.2026.1770152 (PMC13143839; doi:10.3389/fpls.2026.1770152)

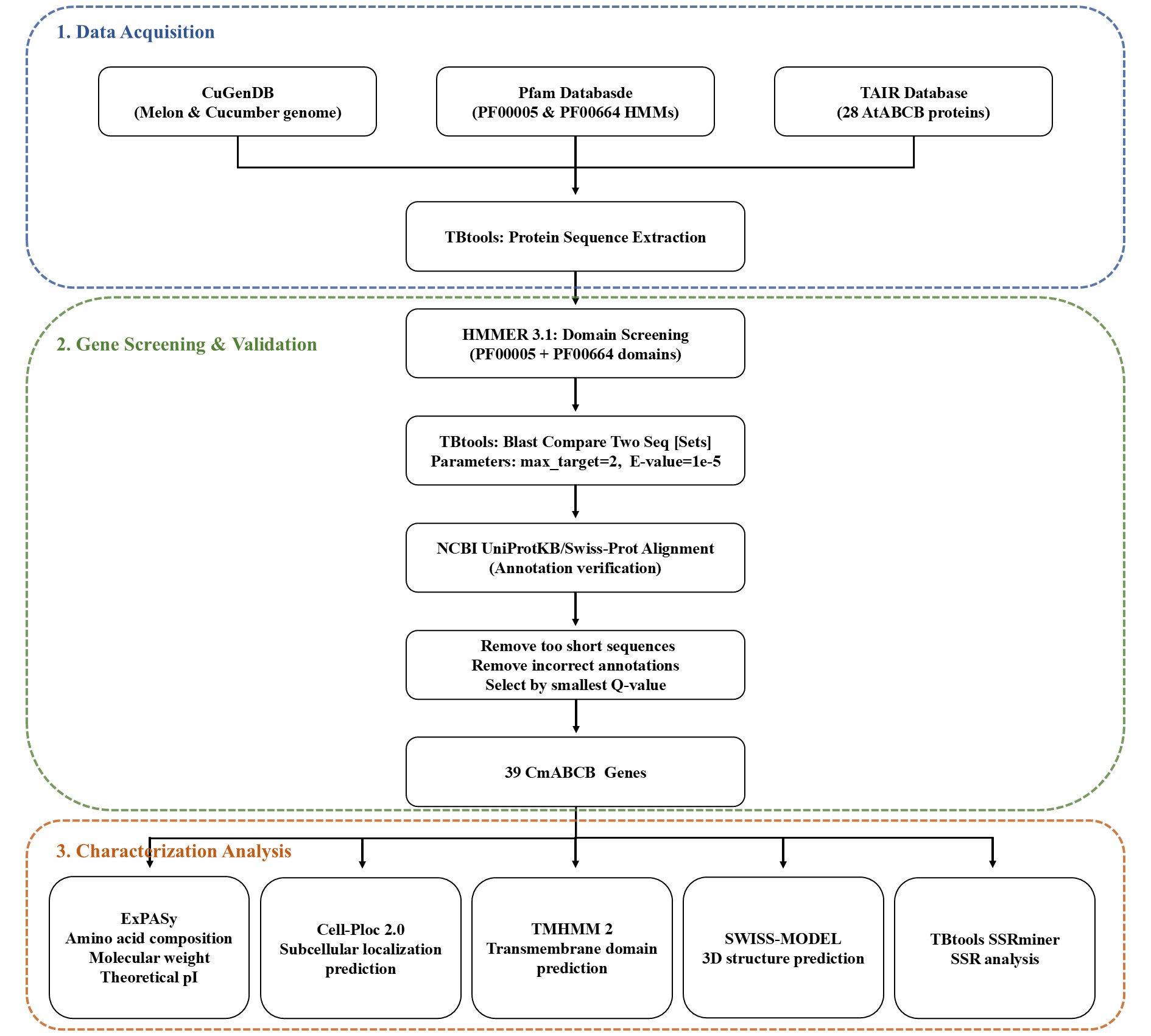

Supplement: Supplementary Figure S1 — Bioinformatics workflow for genome-wide identification and basic characterization of the ABCB gene family in melon (Cucumis melo L.). [file Image1.jpeg]

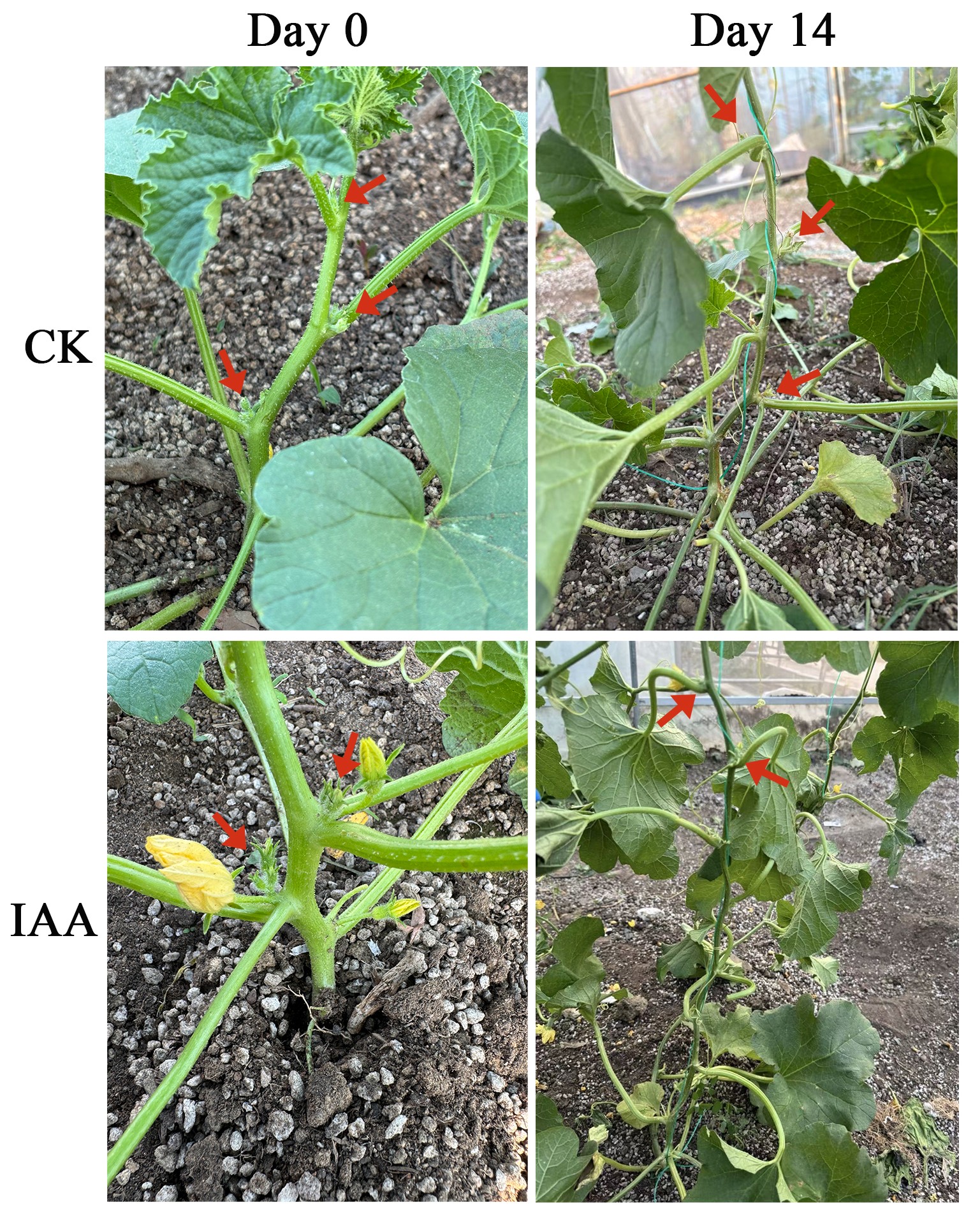

Supplement: Supplementary Figure S2 — Representative phenotypes of melon axillary buds under control (CK) and exogenous IAA treatments at Day 0 and Day 14 (The red arrows in the figure are used to indicate the position of the axillary buds). [file Image2.jpeg]

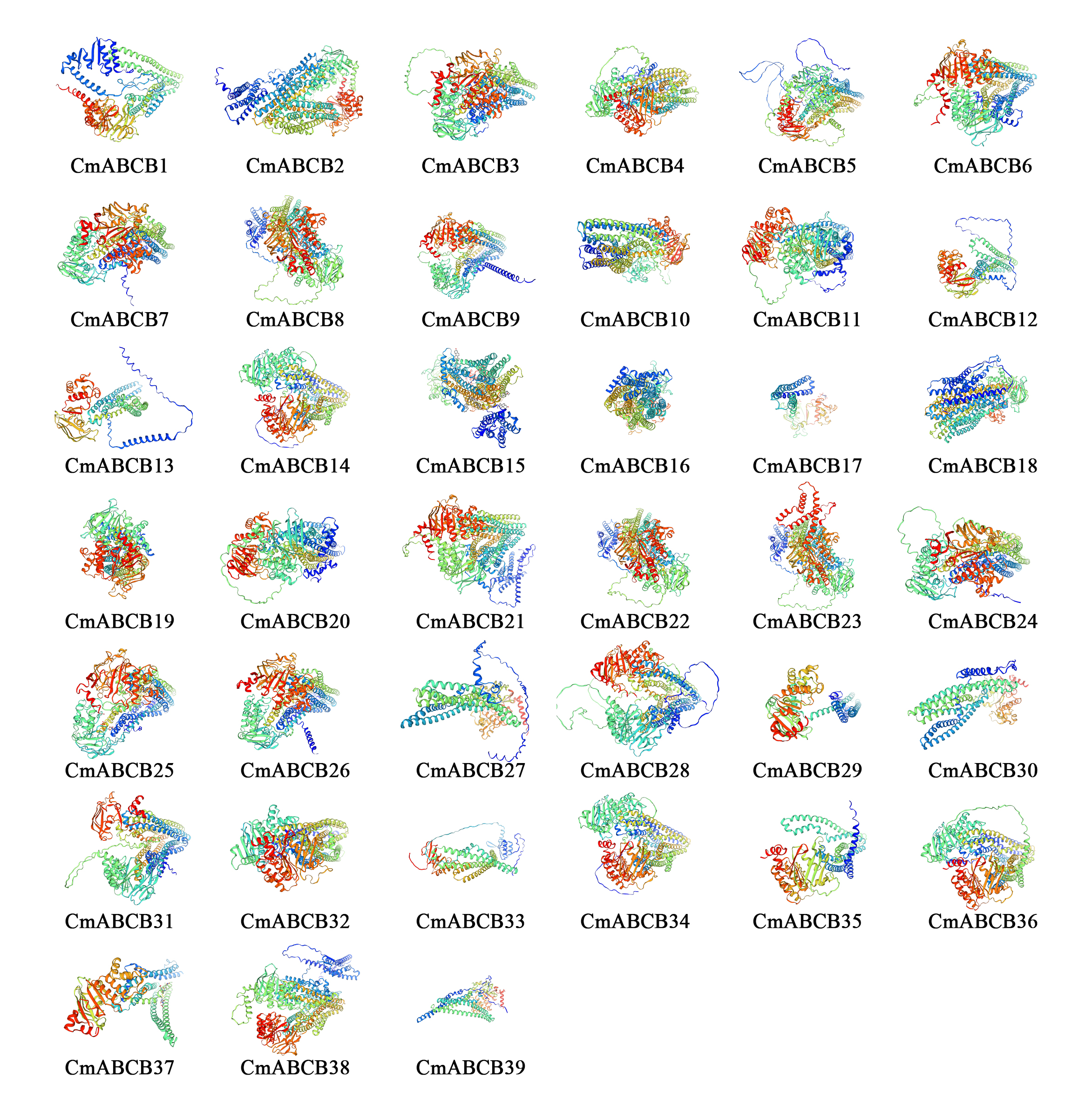

Supplement: Supplementary Figure S3 — Predicted three-dimensional structures of the 39 CmABCB proteins. [file Image3.jpeg]
